# Outcomes Associated With Hospital at Home vs Traditional Inpatient Stay

**DOI:** 10.1001/jamanetworkopen.2026.10810

**Published:** 2026-05-05

**Authors:** J. Priyanka Vakkalanka, Tracy L. Young, Gabriel Bianchi, Fred Ullrich, Knute D. Carter, Nicholas M. Mohr, Jeydith Gutierrez

**Affiliations:** 1 Department of Emergency Medicine, University of Iowa Carver College of Medicine, Iowa City; 2 Department of Epidemiology, University of Iowa College of Public Health, Iowa City; 3 Department of Internal Medicine, University of Iowa Carver College of Medicine, Iowa City; 4 Department of Health Management and Policy, University of Iowa College of Public Health, Iowa City; 5 Department of Biostatistics, University of Iowa College of Public Health, Iowa City; 6 Department of Anesthesia Critical Care, University of Iowa Carver College of Medicine, Iowa City

## Abstract

**Importance:**

Inpatient care is costly, and an aging population, hospital bed shortages, and practitioner shortages stretch inpatient capacity. Alternative modalities of acute care delivery may support growing demands.

**Objective:**

To compare outcomes of hospital at home (HaH) vs traditional inpatient hospital admissions and to assess facility-level variability in HaH utilization.

**Design, Setting, and Participants:**

This propensity score–matched, retrospective, comparative effectiveness research study used data for age-qualifying (≥65 years) fee-for-service Medicare beneficiaries admitted from January 1, 2021, through December 1, 2022, within HaH-waivered US hospitals that had 12 or more HaH admissions. Analyses were completed from November 2024 to March 2026.

**Exposure:**

HaH vs traditional inpatient hospitalization.

**Main Outcomes and Measures:**

Primary clinical outcomes were in-hospital mortality and hospital readmissions and emergency department (ED) visits within 30 days of index admission discharge. Facility-level characteristics were assessed for facilities that had HaH admissions above and below the median (≥149 admissions). Conditional logistic regression was used for dichotomous outcomes, with adjusted odds ratios (aORs) and 95% CIs reported. Log-transformed linear regression was used for skewed continuous outcomes within matched pairs, with adjusted percentage changes and 95% CIs reported.

**Results:**

Among 15 871 Medicare beneficiaries (4174 HaH and 11 697 traditional inpatient admissions), the overall mean (SD) age was 77.4 (8.0) years, and 8396 beneficiaries (56.2%) were female. Of 313 HaH-waivered hospitals, 68 were eligible for inclusion, and 11 hospitals accounted for approximately 50% of all HaH admissions. Compared with traditional inpatient admissions, HaH admissions were associated with lower in-hospital mortality (16 of 4174 admissions [0.4%] vs 423 of 11 697 admissions [3.6%]; aOR, 0.09; 95% CI, 0.06-0.16) and lower ED use within 30 days of discharge (366 of 4174 admissions [8.8%] vs 1164 of 11 697 admissions [10.0%]; aOR, 0.86; 95% CI, 0.76-0.97), with no significant difference in readmissions within 30 days of discharge (490 of 4174 admissions [11.7%] vs 1282 of 11 697 admissions [11.0%]; aOR, 1.07; 95% CI, 0.96-1.20).

**Conclusions and Relevance:**

In this retrospective comparative effectiveness research study of Medicare beneficiaries, HaH was associated with lower in-hospital mortality and ED use within 30 days of discharge, but not hospital readmissions within 30 days, compared with traditional inpatient care. These findings support HaH as an approach that may maintain similar or better short-term outcomes among appropriately selected patients; future studies should evaluate implementation and equity.

## Key Points

**Question:**

How do clinical outcomes of hospital at home compare with traditional inpatient care among Medicare beneficiaries?

**Findings:**

In this propensity score–matched comparative effectiveness research study of 15 871 Medicare beneficiaries (4174 hospital at home and 11 697 traditional inpatient admissions), hospital at home was associated with significantly lower in-hospital mortality and emergency department use within 30 days of index admission discharge, with no significant difference in hospital readmissions within 30 days of index admission discharge compared with traditional inpatient care.

**Meaning:**

These findings suggest that hospital at home was associated with similar or better short-term clinical outcomes compared with traditional inpatient care among appropriately selected patients.

## Teaser

This comparative effectiveness research study compares outcomes of hospital at home vs traditional inpatient hospital admissions and assesses facility-level variability in hospital at home utilization.

## Introduction

Older adults (aged ≥65 years) experience greater hospitalization rates, costs per stay, hospitalization length of stay (LOS), and in-hospital mortality than younger patients.^1^ Older patients are at increased risk for hospital-associated complications, which are common in acute care settings and contribute to clinical deterioration, intensive care unit (ICU) utilization, prolonged hospitalization, and discharge to postacute facilities.^2,3,4,5,6^ Many hospital-associated complications are preventable, highlighting opportunities to improve the safety and efficiency of acute care delivery.^2,3,4,5^ With current and rising strains in health care due to bed and workforce shortages,^7^ there remains a need to evaluate alternative models of care delivery that are clinically effective and reduce costs.

Hospital at home (HaH) is an alternative to traditional inpatient care with the potential to mitigate adverse clinical outcomes and reduce hospital strain. The HaH model refers to the delivery of acute hospital-level care in a patient’s home through a coordinated model, including in-home nursing and clinician visits, diagnostic testing, medication administration, and continuous monitoring.^8^ Since the emergency of HaH in the 1990s,^9^ several early studies showed similar or improved clinical, safety, cost-effectiveness, and/or operational outcomes of HaH models.^10,11,12,13,14^ Despite these promising findings, many of these early studies were conducted in single-center or regional settings, relied on small sample sizes, lacked comparator groups, or were performed before widespread implementation of the model under the Centers for Medicare & Medicaid Services (CMS) Acute Hospital Care at Home (AHCAH) waiver.^15^

Regulatory and government authorities, insurance companies, and health systems seek more compelling evidence to critically appraise the clinical outcomes and costs associated with HaH on a larger, national scale. Although a 2024 report^16^ from CMS comparing HaH and traditional hospitalizations demonstrated improved clinical and process outcomes in the HaH group, the authors identified challenges with this study design, including potentially unbalanced cohorts across critical clinical factors and socioeconomic determinants that could bias the outcomes assessed. That CMS report did not assess hospital-associated complications, a critical outcome among older patients.^16^ We aimed to compare clinical, utilization, and cost outcomes of HaH and traditional inpatient hospitalization among Medicare beneficiaries.

## Methods

### Study Design, Setting, and Sample

We conducted a propensity score–matched, retrospective, comparative effectiveness research study of hospitalized, age-qualifying (≥65 years) Medicare fee-for-service beneficiaries at HaH-waivered hospitals across the US between January 1, 2021, and December 1, 2022. We included all HaH-waivered hospitals that had 12 or more HaH admissions to exclude sites in the early implementation phase. Medicare beneficiary exclusion criteria included those with incomplete calendar year data (owing to late-year enrollment), missing zip codes, and those with extended LOS of more than 42 days. This study was approved by the University of Iowa institutional review board with an expedited review. Informed consent was not required because the research involved deidentified, retrospective administrative data. We report this study in accordance with the Strengthening the Reporting of Observational Studies in Epidemiology (STROBE) reporting guidelines.^17^

### Description of HaH, Data Sources, and Covariates

Under the CMS AHCAH waiver, participating hospitals receive Diagnosis Related Group (DRG)–based inpatient reimbursement with payment parity relative to traditional brick-and-mortar hospitalization. CMS claims data were obtained via the Research Data Assistance Center application process and included a list of HaH-waivered hospitals to identify a cohort of eligible hospitals. All claims for beneficiaries discharged from an HaH-waivered hospital were requested and included inpatient, outpatient, carrier, skilled nursing facility, hospice, home health, and durable medical equipment fee-for-service claims from 2020 to 2022. We used the Master Beneficiary Summary File, with data from Medicare Parts A through D and chronic condition data, and the 2022 American Hospital Association’s annual survey database^18^ to assess hospital-level characteristics (ie, hospital type, urban-rural location, bed capacity, and number of total admissions and discharges among Medicare beneficiaries). Rurality was defined using Rural-Urban Commuting Area code data obtained from the US Department of Agriculture Economic Research Service, and Rural-Urban Commuting Area codes were applied to beneficiary and hospital zip codes for binary classification of urban vs rural location.^19^

### Propensity Score Matching

We modeled the probability of HaH admission using demographic data, clinical covariates, and prior inpatient or emergency department (ED) use, while forcing principal DRG and quarter-year of hospitalization into the model. Beneficiaries with any HaH admission were not eligible as traditional inpatient controls. Each HaH patient was matched to as many as 3 inpatient comparators. Covariate balance was evaluated using standardized differences, with values greater than 0.10 considered imbalanced.

### Outcomes

Primary outcomes were in-hospital mortality (regardless of admission type), readmissions within 30 days of admission discharge, and ED visits within 30 days of admission discharge. Throughout this article, the terms *readmissions* and *ED visits* denote events occurring within 30 days of the discharge date. Secondary outcomes included ICU escalation, hospital-associated complications (eTable 1 in Supplement 1),^20^ and discharge disposition. Continuous outcomes included LOS during the index hospitalization and Medicare-allowed costs from the index admission through 30 days after discharge. Cost outcomes included total costs, total index hospitalization costs, 30-day postdischarge costs, and inpatient readmission costs.

### Statistical Analysis

Analyses were completed from November 2024 to March 2026. We descriptively assessed HaH participant hospitals included in our cohort, and categorized facilities as high vs low utilizers of HaH according to the median number of HaH admissions (median, 149 admissions). This cutoff was used only for descriptive comparisons of hospital characteristics and did not represent a program maturity threshold. We tabulated utilization at the hospital level (above and below this cutoff) by various hospital characteristics from the American Hospital Association. For dichotomous outcomes, we used conditional logistic regression to measure the association between modality of care (ie, HaH vs traditional inpatient admissions) and each outcome within matched patients from the propensity score modeling and reported findings as adjusted odds ratios (aORs) and 95% CIs, with statistical significance defined as a 95% CI that did not include the null value of 1. Since our continuous outcomes (cost and LOS) were skewed, we log-transformed each outcome, used linear regression, stratified within matched pairs, and reported findings as the adjusted percentage change (aPC) associated with HaH and 95% CIs, with statistical significance defined as a 95% CI that did not include the null value of 0. Owing to negligible differences in standardized differences after propensity score matching, final adjusted models included only the modality of hospitalization (ie, HaH vs traditional inpatient). Additional methodological details, including expanded descriptions of data sources, variable definitions, and analytic procedures, are provided in eMethods in Supplement 1. We performed prespecified subgroup and sensitivity analyses stratified by geography, hospital size, census region, and governance.

## Results

### Overview of Hospital Characteristics

Of 313 HaH-waivered hospitals, 68 hospitals met eligibility criteria. Among these 68 hospitals, 11 hospitals accounted for approximately one-half of all HaH admissions. For descriptive comparisons, eligible hospitals were divided at the median (149 admissions) into low-utilizer and high-utilizer groups (Table 1). Most admissions in the 2-year time frame occurred in 2022 (4653 admissions [71.2%]). By geographic region, nearly all high-utilizer hospitals were in the Northeast or South (5 hospitals [45.4%] in each group), with only 1 high-utilizer hospital in the Midwest and none in the West (eFigure 1 in Supplement 1). Nearly all HaH hospitals were urban (62 hospitals [91.2%]), and all 11 high-utilizer hospitals were in urban areas. Most hospitals were nongovernmental or nonprofit institutions (9 hospitals [81.8%] in the high utilizer group and 47 hospitals [82.5%] in the low utilizer group), and the median (IQR) number of total Medicare admissions per year was 28 420 (18 004-44 440) in the high utilizer group compared with 16 887 (9959-26 327) in the low utilizer group.

**Table 1.** Differences in Hospital Characteristics Between Facilities With Low and High HaH Utilization

Characteristics^a^	Hospitals, No. (%)		
	Total	High HaH use^b^	Low HaH use^b^
No. of hospitals	68	11	57
No. of admissions	6537	3261	3276
Total HaH admissions			
2021	1884 (28.8)	1002 (30.7)	882 (26.9)
2022	4653 (71.2)	2259 (69.3)	2394 (73.1)
Geographic location^c^			
Northeast	15 (22.1)	5 (45.4)	10 (17.5)
Midwest	13 (19.1)	1 (9.1)	12 (21.0)
South	29 (42.7)	5 (45.4)	24 (42.1)
West	11 (16.2)	0	11 (19.3)
Rurality			
Urban	62 (91.2)	11 (100.0)	51 (89.5)
Rural	6 (8.8)	0	6 (10.5)
Hospital type			
Nongovernment, not-for-profit	56 (82.3)	9 (81.8)	47 (82.5)
State and local government	12 (17.7)	2 (18.2)	10 (17.5)
Academic affiliation			
No	45 (66.2)	5 (45.4)	40 (70.2)
Yes	23 (33.8)	6 (54.6)	17 (29.8)
System affiliated			
No	7 (10.3)	0	7 (12.3)
Yes	61 (89.7)	11 (100.0)	50 (87.7)
Total staffed beds			
50-99	5 (7.3)	0	5 (8.8)
100-199	12 (17.7)	1 (9.1)	11 (19.3)
200-299	6 (8.8)	0	6 (10.5)
300-399	13 (19.1)	2 (18.2)	11 (19.3)
400-499	7 (10.3)	1 (9.1)	6 (10.5)
>500	25 (36.8)	7 (63.6)	18 (31.6)
Medicare admissions per year, median (IQR)	19 326 (11 054-28 326)	28 420 (18 004-44 440)	16 887 (9959-26 327)
Medicare discharges per year^d^			
<13 376	54 (79.4)	5 (45.4)	49 (86.0)
≥13 376	14 (20.6)	6 (54.6)	8 (14.0)

Abbreviation: HaH, hospital at home.

^a^ Data are from the American Hospital Association 2022 annual survey.^18^

^b^ HaH utilization is based on a cutoff at the median number of enrollments (high, ≥149 enrollments; low, <149 enrollments).

^c^ Northeastern states included Connecticut, Maine, Massachusetts, New Hampshire, New Jersey, New York, Pennsylvania, Rhode Island, and Vermont. Southern states included Alabama, Arkansas, Delaware, District of Columbia, Florida, Georgia, Kentucky, Louisiana, Maryland, Mississippi, North Carolina, Oklahoma, South Carolina, Tennessee, Texas, Virginia, and West Virginia. Midwestern states included Illinois, Indiana, Iowa, Kansas, Michigan, Minnesota, Missouri, Nebraska, North Dakota, Ohio, South Dakota, and Wisconsin. Western states included Montana, Idaho, Wyoming, Colorado, New Mexico, Arizona, Utah, Nevada, Washington, Oregon, California, Alaska, and Hawaii.

^d^ The cutoff of 13 376 Medicare discharges reflects the median annual discharge volume among included hospitals.

### Overview of Sample Characteristics

We initially identified 4383 beneficiaries with an HaH admission and 357 847 beneficiaries with only traditional inpatient admissions as potential controls (eFigure 2 in Supplement 1). Our propensity score–matched sample included 4174 beneficiaries in the HaH cohort and 11 697 matched beneficiaries in the traditional inpatient cohort (15 871 total; mean [SD] age, 77.4 [8.0] years; 8396 female [56.2%]). After propensity score matching, we experienced greater balance in covariates, particularly for age, rurality, and specific clinical comorbid conditions (eg, heart failure, chronic obstructive pulmonary disease, or kidney failure) (Table 2).

**Table 2.** Differences Between Demographic Characteristics and Clinical Outcomes and HaH Utilization Before and After Propensity Score Matching

Characteristics	Before matching			After matching		
	Hospitals and patients, No. (%)		Standardized difference	Hospitals and patients, No. (%)		Standardized difference
	HaH model (n = 4383)	Traditional inpatient (n = 357 847)		HaH model (n = 4174)	Traditional inpatient (n = 11 697)	
Hospital characteristics						
Geographic location^a^						
Northeast	1018 (23.2)	101 609 (28.4)	−0.12	987 (23.7)	2771 (23.7)	<0.01
Midwest	523 (11.9)	63 344 (17.7)	−0.16	502 (12.0)	1466 (12.5)	−0.02
South	2418 (55.2)	140 919 (39.4)	0.32	2266 (54.3)	6177 (52.8)	0.03
West	424 (9.7)	51 975 (14.5)	−0.15	419 (10.0)	1283 (11.0)	−0.03
Rurality						
Urban	4066 (92.8)	342 816 (95.8)	−0.13	3861 (92.5)	10 836 (92.6)	<0.01
Rural	317 (7.2)	15 031 (4.2)	0.13	313 (7.5)	861 (7.4)	<0.01
Hospital type						
Nongovernment, not-for-profit	3585 (81.8)	310 845 (86.9)	−0.14	3401 (81.5)	9497 (81.2)	0.01
State and local government	798 (18.2)	47 002 (13.1)	0.14	773 (18.5)	2200 (18.8)	−0.01
Academic affiliation, yes	2037 (46.5)	172 538 (48.2)	−0.03	1897 (45.4)	5108 (43.7)	0.03
System affiliated, yes	4191 (95.6)	332 936 (93.0)	0.11	3986 (95.5)	11 159 (95.4)	<0.01
Total staffed beds						
50-99	60 (1.4)	6027 (1.7)	−0.02	60 (1.4)	188 (1.6)	−0.02
100-199	613 (14.0)	31 124 (8.7)	0.17	590 (14.1)	1681 (14.4)	−0.01
200-299	325 (7.4)	16 069 (4.5)	0.12	318 (7.6)	957 (8.2)	−0.02
300-399	1035 (23.6)	65 555 (18.3)	0.13	938 (22.5)	2434 (20.8)	0.04
400-499	271 (6.2)	43 160 (12.1)	−0.21	265 (6.4)	749 (6.4)	<0.01
>500	2079 (47.4)	195 912 (54.8)	−0.15	2003 (48.0)	5688 (48.6)	−0.01
No. of Medicare admissions per year						
Mean (SD)	26 013 (14 941)	29 335 (15 750)	−0.22	26 165 (15 082)	26 342 (15 337)	−0.01
Median (IQR)	22 281 (16 536 to 36 042)	25 887 (17 299 to 39 334)		22 972 (15 179 to 36 042)	22 972 (13 860 to 37 606)	
No. of Medicare discharges per year						
Mean (SD)	11 226 (5646)	12 209 (5868)	−0.17	11 288 (5698)	11 385 (5830)	−0.02
Median (IQR)	9034 (6959 to 16 343)	10 613 (8231 to 16 343)		9034 (6959 to 16 343)	9819 (6626 to 16 343)	
Patient demographic characteristics						
Age, y						
65-74	1676 (38.2)	154 549 (43.2)	−0.10	1591 (38.1)	4490 (38.4)	−0.01
75-84	1722 (39.3)	132 477 (37.0)	0.05	1632 (39.1)	4466 (38.2)	0.02
≥85	985 (22.5)	70 821 (19.8)	0.07	951 (22.8)	2741 (23.4)	−0.02
Sex						
Female	2293 (52.3)	187 401 (52.4)	<0.01	2202 (52.8)	6194 (53.0)	<0.01
Male	2090 (47.7)	170 446 (47.6)		1972 (47.2)	5503 (47.0)	
Race and ethnicity^b^						
Asian	53 (1.2)	4399 (1.2)	<0.01	51 (1.2)	146 (1.2)	<0.01
Black	282 (6.4)	23 798 (6.7)	−0.01	271 (6.5)	781 (6.7)	−0.01
Hispanic	57 (1.3)	3860 (1.1)	0.02	55 (1.3)	182 (1.6)	−0.02
White	3858 (88.0)	312 060 (87.2)	0.02	3668 (87.9)	10 258 (87.7)	0.01
Other	56 (1.3)	6236 (1.7)	−0.03	55 (1.3)	147 (1.3)	0.01
Unknown	77 (1.8)	7494 (2.1)	−0.02	74 (1.8)	183 (1.6)	0.02
Rurality						
Urban	4245 (96.9)	316 386 (88.4)	0.33	4037 (96.7)	11 310 (96.7)	<0.01
Rural	138 (3.1)	41 461 (11.6)	−0.33	137 (3.3)	387 (3.3)	<0.01
Patient clinical characteristics						
Prior emergency department utilization, yes	2129 (48.6)	106 049 (29.6)	0.40	1990 (47.7)	5472 (46.8)	0.02
Prior hospitalization, yes	1914 (43.7)	62 878 (17.6)	0.59	1752 (42.0)	4656 (39.8)	0.04
Select Elixhauser comorbid conditions^c^						
Anemias due to nutritional deficiencies	1073 (24.5)	66 614 (18.6)	0.14	1002 (24.0)	2755 (23.6)	0.01
Autoimmune conditions	317 (7.2)	18 078 (5.0)	0.09	300 (7.2)	819 (7.0)	0.01
Metastatic cancer	147 (3.3)	14 940 (4.2)	−0.05	140 (3.3)	376 (3.2)	0.01
Solid tumor without metastasis, malignant	266 (6.1)	16 010 (4.5)	0.07	251 (6.0)	641 (5.5)	0.02
Cerebrovascular disease	169 (3.9)	24 518 (6.9)	−0.13	164 (3.9)	473 (4.0)	−0.01
Heart failure	1524 (34.8)	89 999 (25.1)	0.21	1458 (34.9)	4169 (35.6)	−0.01
Coagulopathy	397 (9.1)	26 497 (7.4)	0.06	369 (8.8)	999 (8.5)	0.01
Dementia	350 (8.0)	40 121 (11.2)	−0.11	346 (8.3)	985 (8.4)	<0.01
Depression	643 (14.7)	49 764 (13.9)	0.02	608 (14.6)	1762 (15.1)	−0.01
Diabetes without chronic complications	410 (9.3)	39 329 (11.0)	−0.06	399 (9.6)	1155 (9.9)	−0.01
Diabetes with chronic complications	1142 (26.1)	74 740 (20.9)	0.12	1080 (25.9)	2955 (25.3)	0.01
Hypertension, complicated	1555 (35.5)	118 917 (33.2)	0.05	1469 (35.2)	4052 (34.6)	0.01
Hypertension, uncomplicated	1586 (36.2)	155 147 (43.4)	−0.15	1516 (36.3)	4225 (36.1)	<0.01
Liver disease, mild	194 (4.4)	14 569 (4.1)	0.01	188 (4.5)	501 (4.3)	0.01
Chronic pulmonary disease	1425 (32.5)	79 717 (22.3)	0.23	1356 (32.5)	3797 (32.5)	<0.01
Neurological disorders affecting movement	249 (5.7)	16 463 (4.6)	0.05	239 (5.7)	657 (5.6)	<0.01
Other neurological disorders	274 (6.2)	34 481 (9.6)	−0.13	268 (6.4)	815 (7.0)	−0.02
Obesity	849 (19.4)	62 099 (17.3)	0.05	814 (19.5)	2276 (19.5)	<0.01
Peripheral vascular disease	389 (8.9)	35 463 (9.9)	−0.03	367 (8.8)	1044 (8.9)	<0.01
Pulmonary circulation disease	354 (8.1)	20 465 (5.7)	0.09	340 (8.1)	986 (8.4)	−0.01
Kidney failure, moderate	1086 (24.8)	67 192 (18.8)	0.15	1021 (24.5)	2861 (24.5)	<0.01
Kidney failure, severe	274 (6.2)	20 750 (5.8)	0.02	265 (6.3)	720 (6.2)	0.01
Hypothyroidism	1028 (23.4)	75 012 (21.0)	0.06	972 (23.3)	2671 (22.8)	0.01
Valvular disease	638 (14.6)	47 683 (13.3)	0.04	604 (14.5)	1737 (14.9)	−0.01
Weight loss	334 (7.6)	29 689 (8.3)	−0.03	316 (7.6)	880 (7.5)	<0.01

Abbreviation: HaH, hospital at home.

^a^ Northeastern states included Connecticut, Maine, Massachusetts, New Hampshire, New Jersey, New York, Pennsylvania, Rhode Island, and Vermont. Southern states included Alabama, Arkansas, Delaware, District of Columbia, Florida, Georgia, Kentucky, Louisiana, Maryland, Mississippi, North Carolina, Oklahoma, South Carolina, Tennessee, Texas, Virginia, and West Virginia. Midwestern states included Illinois, Indiana, Iowa, Kansas, Michigan, Minnesota, Missouri, Nebraska, North Dakota, Ohio, South Dakota, and Wisconsin. Western states included Montana, Idaho, Wyoming, Colorado, New Mexico, Arizona, Utah, Nevada, Washington, Oregon, California, Alaska, and Hawaii.

^b^ The other category includes race and ethnicity groups not reported separately due to small cell sizes, consistent with Centers for Medicare & Medicaid Services data use requirement.

^c^ Of the 38 Elixhauser comorbidities from the 2025 Healthcare Cost & Utilization Project, we report on conditions that were present in at least 3% of the sample.

### Overview of Outcomes

We observed 2.8% in-hospital mortality (439 patients), 11.2% readmission (1772 patients), and 9.6% return to the ED (1530 patients) (Table 3). In addition, 6.7% of patients (1070 patients) experienced an escalation of care to the ICU, 4.7% (752 patients) developed hospital-associated complications, and 78.7% (12 138 patients) were discharged home. The median (IQR) LOS was 5 (4-8) days, the median (IQR) total health care costs were $17 074 ($11 554-$26 595), the median (IQR) index hospitalization costs were $12 128 ($8745-$17 132), the median (IQR) inpatient readmission costs were $14 138 ($10 039-$22 095), and the median (IQR) postdischarge costs were $2955 ($800-$6375).

**Table 3.** Summary of Outcomes by HaH Utilization

Outcomes	Patients, No. (%)			RD, % (95% CI)	Standardized difference
	Total (N = 15 871)	HaH patients (n = 4174)	Matched cohort (n = 11 697)		
Dichotomous outcomes					
In-hospital mortality^a^	439 (2.8)	16 (0.4)	423 (3.6)	−3.20 (−3.59 to −2.81)	NA
30-d Readmission^a^	1772 (11.2)	490 (11.7)	1282 (11.0)	0.70 (−0.43 to 1.83)	NA
Emergency department visits 30 d after discharge^a^	1530 (9.6)	366 (8.8)	1164 (10.0)	−1.20 (−2.22 to −0.18)	NA
Intensive care unit escalation	1070 (6.7)	145 (3.5)	925 (7.9)	−4.40 (−5.14 to −3.66)	NA
Hospital-related condition^b^	752 (4.7)	150 (3.6)	602 (5.1)	−1.50 (−2.19 to −0.81)	NA
Discharge location					
Home	12 138 (78.7)	3945 (94.9)	8193 (72.7)	22.20 (21.15 to 23.25)	NA
Rehabilitation	2369 (15.3)	88 (2.1)	2281 (20.2)	−18.10 (−18.95 to −17.25)	NA
Hospice	469 (3.0)	53 (1.3)	416 (3.7)	−2.40 (−2.89 to −1.92)	NA
Other	456 (3.0)	72 (1.7)	384 (3.4)	−1.70 (−2.21 to −1.19)	NA
Continuous outcomes					
Length of index hospitalization					
No.	15 871	4174	11 697	NA	1.12
Mean (SD), d	6.53 (4.66)	7.36 (4.76)	6.24 (4.59)	NA	
Median (IQR), d	5.00 (4.00 to 8.00)	6.00 (4.00 to 9.00)	5.00 (3.00 to 8.00)	NA	
Total health care costs, $^c^					
No.	15 846	4172	11 674	NA	<0.01
Mean (SD)	21 798 (17 072)	20 114 (14 549)	22 399 (17 849)	NA	
Median (IQR)	17 074 (11 554 to 26 595)	16 262 (11 420 to 23 386)	17 424 (11 607 to 27 661)	NA	
Total index hospitalization costs, $^c^					
No.	15 736	4164	11 572	NA	0.03
Mean (SD)	14 492 (10 715)	14 643 (9556)	14 437 (11 102)	NA	
Median (IQR)	12 128 (8745 to 17 132)	12 561 (9154 to 17 426)	11 972 (8624 to 17 035)	NA	
Total inpatient readmission costs, $^c^					
No.	1758	490	1268	NA	<0.01
Mean (SD)	19 643 (17 965)	17 788 (15 374)	20 359 (18 828)	NA	
Median (IQR)	14 138 (10 039 to 22 095)	13 672 (9858 to 20 240)	14 298 (10 173 to 23 397)	NA	
Total postdischarge costs, $^c^					
No.	14 717	4021	10 696	NA	<0.01
Mean (SD)	$5629 ($7977)	$3538 ($4767)	$6414 ($8761)	NA	
Median (IQR)	$2955 ($800 to $6375)	$2293 ($618 to $4167)	$3199 ($917 to $7858)	NA	

Abbreviations: HaH, hospital at home; NA, not applicable; RD, risk difference.

^a^ Denotes primary outcomes.

^b^ Hospital-related conditions included stage III or IV pressure ulcers, falls or trauma, poor glycemic control, catheter-associated urinary tract infection, vascular catheter-associated infection, deep vein thrombosis or pulmonary embolism following certain orthopedic procedures, and iatrogenic pneumothorax with venous catheterization.

^c^ All cost components include Medicare payment, primary payer payment, and beneficiary payment adjusted to 2022 US dollars.

### Primary Clinical Outcomes: In-Hospital Mortality, Readmissions, and ED Utilization

In the matched cohort, outcomes for HaH admissions compared with traditional inpatient admissions included the following: in-hospital mortality (16 of 4174 admissions [0.4%] vs 423 of 11 697 admissions [3.6%]), readmissions for 1158 individuals (490 of 4174 admissions [11.7%] vs 1282 of 11 697 admissions [11.0%]), and ED visits (366 of 4174 admissions [8.8%] vs 1164 of 11 697 admissions [10.0%]) (Table 3). Compared with the traditional inpatient group, HaH patients had significantly lower odds of in-hospital mortality (aOR, 0.09; 95% CI, 0.06-0.16) and ED utilization (aOR, 0.86; 95% CI, 0.76-0.97); however, we did not observe any differences in hospital readmission (aOR, 1.07; 95% CI, 0.96-1.20) (Table 4).

**Table 4.** Associations Between Hospital at Home Utilization and Clinical, Operational, and Economic Outcomes

Outcomes	aOR (95% CI)^a^
Primary outcomes (dichotomous)	
Mortality during hospitalization	0.09 (0.06-0.16)
Readmission within 30 d of discharge	1.07 (0.96-1.20)
Emergency department visits within 30 d of discharge	0.86 (0.76-0.97)
Secondary outcomes (dichotomous)	
Intensive care unit escalations	0.39 (0.33-0.48)
Hospital-associated complications	0.59 (0.48-0.73)
Disposition	
Home	8.02 (6.93-9.29)
Rehabilitation	0.09 (0.07-0.11)
Hospice	0.34 (0.25-0.45)
Other locations	0.53 (0.41-0.68)
Secondary outcomes (continuous)	
Hospital length of stay	1.23 (1.21-1.26)^b^
Costs	
Total health care costs	0.96 (0.94-0.98)^b^
Index hospitalization costs	1.10 (1.08-1.12)^b^
Postdischarge costs (within 30 d)	0.65 (0.62-0.68)^b^
Inpatient readmission costs (within 30 d)	0.97 (0.90-1.04)^b^

Abbreviation: aOR, adjusted odds ratio.

^a^ The reference value is traditional inpatient admission.

^b^ Data are adjusted percentage change (95% CI).

### Secondary Outcomes: Need for ICU Escalation, Development of Hospital-Associated Complications, and Disposition

In the matched cohort, outcomes for HaH admissions compared with traditional inpatient admissions included the following: need for ICU escalation (145 of 4174 patients [3.5%] vs 925 of 11 697 patients [7.9%]), development of hospital-associated complications (150 of 4174 patients [3.6%] vs 602 of 11 697 patients [5.1%]), and disposition to home (3945 of 4174 patients [94.9%] vs 8193 of 11 697 patients [72.7%]) (Table 3). Compared with the traditional inpatient group, HaH patients had lower odds of escalation of care to ICU (aOR, 0.39; 95% CI, 0.33-0.48) and development of hospital-associated complications (aOR, 0.59; 95% CI, 0.48-0.73). Disposition to home was greater in the HaH cohort (aOR, 8.02; 95% CI, 6.93-9.29), whereas disposition to rehabilitation center (aOR, 0.09; 95% CI, 0.07-0.11), hospice (aOR, 0.34; 95% CI, 0.25-0.45), and other types of care (aOR, 0.53; 95% CI, 0.41-0.68) were lower for patients with an HaH admission (Table 4).

### Secondary Outcomes: LOS and Costs

We observed an increase in index admission LOS in the HaH group (aPC, 1.23%; 95% CI, 1.21%-1.26%) (Table 4). Cost outcomes differed by phase of care. The HaH group showed minor decreases in total health care costs (aPC, 0.96%; 95% CI, 0.94%-0.98%). Within the subcomponents of total costs, we observed increased total index hospitalization costs (aPC, 1.10%; 95% CI, 1.08%-1.12%) yet a decrease in total discharge costs within 30 days (aPC, 0.65%; 95% CI, 0.62%-0.68%). Readmission costs among the 1758 patients with cost data (of the 1772 with a readmission) were not significantly different (aPC, 0.97%; 95% CI, 0.90%-1.04%).

### Sensitivity and Subgroup Analyses

When we evaluated baseline severity using a claims-based prognostic model that excluded admission modality (ie, HaH vs traditional inpatient), the estimated risk of in-hospital mortality was nearly identical across groups (3.3% for traditional inpatient vs 3.0% for HaH; absolute difference, 0.3%). In subgroup and sensitivity analyses, results were directionally consistent with the primary estimates with some variability in magnitude (eTables 2-7 in Supplement 1). For example, we observed some variability for readmissions, which were higher in rural hospitals and in the South, with a borderline increase in smaller hospitals (eTables 2, 3, and 6 in Supplement 1).

## Discussion

In this national comparative effectiveness research study, HaH demonstrated similar or better short-term clinical outcomes and comparable costs vs traditional inpatient hospitalizations. These findings likely reflect outcomes during early implementation of HaH where clinicians enrolled clinically and operationally appropriate patients for the HaH mechanism. At the hospital level, HaH admissions were highly concentrated within a subset of facilities, particularly in the US Northeast and South. In aggregate, these findings suggest that HaH can deliver safe, efficient, alternative acute care while alleviating pressures on health care systems. Second, they underscore the need to address practical and implementation challenges to broaden equitable access and obtain consistent clinical benefits across health systems.

Our primary findings are consistent with prior findings, including a systematic review of randomized clinical trials by Arsenault-Lapierre et al^21^ that found HaH was associated with similar or lower mortality, although with reduced readmissions. Similarly, in a systematic review by Sultani et al,^22^ HaH was found to be at least as safe as usual care and with lower costs, but with longer LOS. In contrast to national descriptive reports of the CMS AHCAH experience,^16^ our analysis incorporates a matched comparator cohort and expanded outcome assessment, enabling evaluation of clinical safety, care transitions, and downstream utilization among patients selected for HaH. From this study, we provide evidence that is complementary to federal and congressional summaries^23^ by advancing the methods to estimate a comparative effectiveness of the HaH and traditional inpatient models.

The primary clinical outcomes examined in this study align with those of previous randomized clinical trials, including 1 conducted within rural areas^24^ and 1 in a single-system setting,^13^ demonstrating that substitutive home hospital care was associated with reduced costs, decreased health care use, improved physical activity, and decreased 30-day readmissions compared with usual hospital care. More recently, a pragmatic multicenter trial^25^ comparing a virtual-hybrid HaH model with traditional inpatient care demonstrated noninferior 30-day outcomes (including a mortality and unplanned readmissions composite outcome), further supporting the safety of newer HaH models. Within the rural trial by Levine et al,^24^ late transfers to the home setting could have attenuated observed utilization and cost differences. This may explain some of the heterogeneity we observed across facilities in our sample. In addition, a descriptive study by Levine et al^26^ of a similar cohort of Medicare beneficiaries that extended beyond our study window after the peak of the COVID-19 pandemic reflected similar low rates of adverse outcomes and health care utilization after admission. When combined with our analytical findings, the results provide a more complete picture of the evolution and outcomes of HaH under the AHCAH waiver. Prior work evaluating transfer to HaH programs has also shown consistent results; for instance, Cai and colleagues^27^ found that veterans experienced more days at home and reduced postacute facility use without increased costs and with similar mortality among those discharged alive. These findings are also consistent with our observation that shorter-term postdischarge outcomes were not worse with HaH.

Previous studies identified lower costs associated with HaH, and contributing factors may include shorter LOS and fewer diagnostics. In our study, index hospitalization costs were modestly higher for HaH; however, postdischarge spending (including ED use and postacute services) was lower, yielding slightly lower total 30-day episode costs. However, the direction and magnitude of differences in costs may depend on the model design, including whether HaH is a substitutive vs step-down approach, as well as reimbursement patterns related to hospital type. Although we controlled for DRG, patients in HaH and traditional inpatient settings could have originated from hospitals with similar observable characteristics but different payment adjustments. Although Medical Severity–DRG weights are standardized, final payments vary on the basis of geographic wage indices, teaching status, disproportionate share payments, and other facility level adjustments. As a result, matching on DRG does not fully account for hospital-level payment differences. Forcing facilities into the matching process could have reduced this variation, but would have substantially limited the availability of suitable controls. Although cost comparisons should be interpreted cautiously, we did not account for system-level cost avoidance; removing more acute patients from HaH eligibility allows hospitals to backfill beds with patients who generate higher revenue, which could shift the financial impact at the system level. Our findings focus on direct episode spending and do not capture these broader economic considerations.

Our observation of lower hospital-associated complications in HaH is concordant with mechanistic literature linking reduced exposure to ward-based care with fewer adverse events.^28,29,30,31^ Prior studies have demonstrated that each additional inpatient day increases the risk of adverse drug reactions, infections, and pressure ulcers.^32^ HaH programs reduce the time spent in the hospital environment and may, therefore, decrease opportunities for hospital-associated complications. Despite these advantages, national adoption of HaH is uneven, with limited use in rural settings and the highest adoption in larger academic health centers. Qualitative work among rural clinicians and patients has similarly highlighted substantial barriers to implementing HaH in rural settings, including geographic distances, broadband limitations, staffing constraints, and local hospital politics.^33^ Such context-specific challenges may contribute to the lower HaH adoption in our sample. As previous work has also indicated, there are several implementation factors and logistical barriers with the current HaH model at the patient (eg, buy-in, patient selection, and triage),^34,35,36,37^ system (eg, technological and collaborations),^34,35,38,39^ and societal (eg, policy, regulatory, and payment) levels.^34,39,40^ Program variability may contribute to differences in outcomes and scalability; DeCherrie et al^41^ describe the evolution of a single urban HaH program into an additive model that included, but was not limited to, palliative care, observation-level care, and postacute rehabilitation. Similarly, a randomized clinical trial^42^ comparing remote vs in-home physician visits during HaH care demonstrated noninferior safety and patient experience, suggesting that alternative physician-visit models may be feasible, although a subset of patients still required in-person evaluation. This highlights the operational trade-offs health systems face when scaling HaH programs, and the heterogeneity may explain some of our observed variability. Qualitative national work further highlights staffing, logistics, and cross-sector coordination as persistent barriers; addressing these constraints is essential to broaden access and realize consistent outcomes.

### Limitations

There are limitations that should be mentioned. This observational analysis used Medicare fee-for-service claims, which limits causal inference and may incompletely capture clinical acuity and context. Residual selection bias is possible despite propensity score matching and good balance on measured covariates, because claims lack key eligibility and triage factors, such as physiological severity at presentation, functional and cognitive status, caregiver support, home suitability, and patient preference. The findings should, therefore, be interpreted as associations among patients selected for HaH. Claims do not routinely capture geographic eligibility criteria for HaH, including distance to the hospital and program catchment areas, so we could not adjust for these factors. The study period reflects early US experience under the CMS AHCAH waiver during and immediately after the COVID-19 public health emergency. Program maturity and pandemic-era operational pressures may have influenced patient selection, implementation, and outcomes. Newer National Uniform Billing Committee span codes adopted after mid-2022 may enable clearer differentiation of HaH days within an acute episode in future work; however, we ensured that no HaH admissions followed a traditional inpatient admission. Small counts required pooling some discharge dispositions; skilled nursing facility discharges were combined within a broader postacute category to meet statistical stability and data privacy requirements. In addition, our descriptive comparison of higher vs lower HaH utilization used a data-driven threshold to preserve sample sizes and should not be interpreted as program maturity or quality.

## Conclusions

HaH was associated with lower in-hospital mortality and ED use within 30 days after discharge, but not hospital readmissions within 30 days after discharge, compared with traditional inpatient care. Although index hospitalization costs were higher for HaH, lower postdischarge costs contributed to modest reductions in total 30-day health care spending, highlighting potential economic advantages without compromising short-term clinical outcomes.

## Notes

Supplement 1. eMethods. Expanded Description of Methodology eTable 1. Hospital-Related Conditions and *ICD-10-CM* Codes (Centers for Medicare and Medicaid/CMS) eTable 2. Subgroup Analysis: Main Analysis, Rural Hospitals, and Urban Hospitals eTable 3. Subgroup Analysis: Main Analysis, Hospitals with ≥200 Beds, and Hospitals with ≤199 Beds eTable 4. Subgroup Analysis: Main Analysis and 2022 Admissions Only eTable 5. Subgroup Analysis: Main Analysis and Admissions Excluding Arrivals from Nursing Facilities eTable 6. Subgroup Analysis: Main Analysis, Hospitals in US Northeast, and Hospitals in US South eTable 7. Subgroup Analysis: Main Analysis, Non-government Hospitals, and State/Local Hospitals eFigure 1. Hospital at Home Admissions by Facility in the US (2021–2022) eFigure 2. Flowchart of Sample Selection and Final Analytical Cohort (Medicare, 2021—2022) Before Propensity Score Matching

Supplement 2. Data Sharing Statement
