# Supplementary material for: Outcomes Associated With Hospital at Home vs Traditional Inpatient Stay
Source: JAMA Netw Open. 2026 May 5;9(5):e2610810. doi: 10.1001/jamanetworkopen.2026.10810 (PMC13147191; doi:10.1001/jamanetworkopen.2026.10810)
Supplement: Supplement 1. — eMethods. Expanded Description of Methodology eTable 1. Hospital-Related Conditions and ICD-10-CM Codes (Centers for Medicare and Medicaid/CMS) eTable 2. Subgroup Analysis: Main Analysis, Rural Hospitals, and Urban Hospitals eTable 3. Subgroup Analysis: Main Analysis, Hospitals with ≥200 Beds, and Hospitals with ≤199 Beds eTable 4. Subgroup Analysis: Main Analysis and 2022 Admissions Only eTable 5. Subgroup Analysis: Main Analysis and Admissions Excluding Arrivals from Nursing Facilities eTable 6. Subgroup Analysis: Main Analysis, Hospitals in US Northeast, and Hospitals in US South eTable 7. Subgroup Analysis: Main Analysis, Non-government Hospitals, and State/Local Hospitals eFigure 1. Hospital at Home Admissions by Facility in the US (2021–2022) eFigure 2. Flowchart of Sample Selection and Final Analytical Cohort (Medicare, 2021—2022) Before Propensity Score Matching [file jamanetwopen-e2610810-s001.pdf]

Supplemental Online Content

Vakkalanka JP, Young TL, Bianchi G, et al. Outcomes associated with hospital at home vs traditional inpatient stay. *JAMA Netw Open*. 2026;9(5):e2610810. doi:10.1001/jamanetworkopen.2026.10810

eMethods. Expanded Description of Methodology
eTable 1. Hospital-related conditions and *ICD-10-CM* codes (Centers for Medicare and Medicaid/CMS)
eTable 2. Subgroup Analysis: Main Analysis, Rural Hospitals, and Urban Hospitals
eTable 3. Subgroup Analysis: Main Analysis, Hospitals with ≥200 Beds, and Hospitals with ≤199 Beds
eTable 4. Subgroup Analysis: Main Analysis and 2022 Admissions Only
eTable 5. Subgroup Analysis: Main Analysis and Admissions Excluding Arrivals from Nursing Facilities
eTable 6. Subgroup Analysis: Main Analysis, Hospitals in US Northeast, and Hospitals in US South
eTable 7. Subgroup Analysis: Main Analysis, Non-government Hospitals, and State/Local Hospitals
eFigure 1. Hospital at Home Admissions by Facility in the US (2021–2022)
eFigure 2. Flowchart of Sample Selection and Final Analytical Cohort (Medicare, 2021—2022) Before Propensity Score Matching

This supplemental material has been provided by the authors to give readers additional information about their work.

© 2026 Vakkalanka JP et al. *JAMA Network Open*.

eMethods. Expanded Description of Methodology

Study Design, Setting, and Sample. We conducted a propensity-score matched retrospective cohort study of hospitalized, age qualifying (□65 years) Medicare fee-for-service beneficiaries at HaH-waivered hospitals across the US between January 1, 2021 and December 1, 2022. We included all HaH-waivered hospitals that had at least 12 HaH admissions during the study period to exclude sites in the very earliest implementation phase. To minimize confounding by hospital-level factors, we restricted the comparator group to traditional inpatient admissions from the same hospitals that were HaH-waivered. This approach ensured that both groups were drawn from settings with similar organizational structures, admitting pathways, and access to HaH infrastructure. Because Medicare claims lack detailed clinical and social data that determine HaH eligibility (e.g., physiologic severity, functional status, home environment, caregiver support, and patient preference), propensity score matching was limited to measured demographic characteristics, comorbidities, diagnosis related groups, and prior utilization.

Medicare beneficiary exclusion criteria included those with incomplete calendar year data (due to late-year enrollment), missing zip codes, Medicare Advantage enrollees, and those with extended lengths of stay of >42 days. This study was approved by the University of Iowa Institutional Review Board with expedited review. Informed consent was not required because the research involved de-identified, retrospective administrative data. We report this study in accordance with the Strengthening the Reporting of Observational Studies in Epidemiology (STROBE) guidelines.[18]

© 2026 Vakkalanka JP et al. *JAMA Network Open*.

Description of HaH. Under the CMS Acute Hospital Care at Home waiver, participating hospitals receive Diagnosis Related Group- (DRG) based inpatient reimbursement with payment parity relative to traditional brick-and-mortar hospitalization. Hospitals remain financially responsible for delivering all required hospital-level services in the home, including nursing care, clinician evaluation, diagnostics, medication administration, and remote monitoring. Although reimbursement is aligned with inpatient payment, services may be billed across multiple Medicare claim types rather than a single institutional claim.

Data Sources and Covariates. CMS claims data were obtained via the Research Data Assistance Center (ResDAC) application process and included a list of HaH-waivered hospitals to identify a cohort of eligible hospitals. All claims for beneficiaries (regardless of HaH participation) discharged from an HaH hospital were requested and included inpatient, outpatient, carrier, skilled nursing facility, hospice, home health, and durable medical equipment fee-for-service claims from 2020 to 2022. In addition, we used the Master Beneficiary Summary File (MBSF) base (A/B/C/D) and chronic condition data. Finally, we utilized the 2022 American Hospital Association's (AHA) annual survey database to assess hospital-level characteristics of the HaH-participating sites. These included hospital type (e.g., ownership, pay structure, teaching vs community), urban-rural location, bed capacity, and number of total admissions and discharges among Medicare beneficiaries. Rurality was defined using Rural–Urban Commuting Area (RUCA) codes and applied to beneficiary and hospital ZIP codes. For this analysis, RUCA codes were grouped into a binary classification of urban versus rural, obtained from the USDA Economic Research Service,[19] to support descriptive comparisons of

© 2026 Vakkalanka JP et al. *JAMA Network Open*.

hospital characteristics and to maintain adequate cell sizes. Urban areas included RUCA codes 1.0, 1.1, 2.0, 2.1, 3.0, 4.1, 5.1, 7.1, 8.1, and 10.1, while rural areas included RUCA codes 4.0, 4.2, 5.0, 5.2, 6.0, 6.1, 7.0, 7.2, 7.3, 7.4, 8.0, 8.2, 8.3, 8.4, 9.0, 9.1, 9.2, 10.0, 10.2, 10.3, 10.4, 10.5, and 10.6.

Propensity Score Matching. From the hospital sample, we identified patients who had a HaH admission and those with a traditional inpatient visit. Patients who had at least one HaH hospitalization were excluded from selection as traditional inpatient controls. Because claims do not capture all eligibility criteria for HaH, we emphasize that propensity score methods balance measured covariates but cannot account for unmeasured clinical and social factors influencing selection. We first modeled the propensity for an HaH admission using the following covariates: beneficiary age, demographic characteristics (age, sex, race/ethnicity, rurality), Elixhauser comorbid conditions, hospital, and prior year inpatient admissions and emergency department (ED) visits. We additionally forced principal admission using DRG groups from Medicare claims and time of hospitalization (quarter/year) to account for temporal variability as matching criteria in the propensity score model. Race and ethnicity were obtained from the Medicare Limited Data Set and were originally reported by beneficiaries to the Social Security Administration and incorporated into Medicare administrative records. Categories were reported as Asian, Black, Hispanic, White, Other, and Unknown, consistent with available Medicare data. We matched each HaH beneficiary (i.e., exposed) with up to 3 traditional inpatient beneficiaries (i.e., unexposed). To assess balance of covariates between both treatment groups before and after propensity score

© 2026 Vakkalanka JP et al. *JAMA Network Open*.

matching, we determined *a priori* that covariates with a standardized difference >0.10 would be considered for multivariable modeling.

Outcomes. Primary outcomes were in-hospital mortality (regardless of admission type), readmissions within 30 days of admission discharge, and ED visits within 30 days of admission discharge. For readmissions, observation stays were not included because they are billed separately and cannot be consistently linked to the hospitalization episode in this dataset. We measured several clinical and process-related secondary outcomes during the hospitalization (between admission and discharge dates) and within 30-days of hospital discharge. Dichotomous outcomes included escalation of care to the ICU, development of hospital-associated complications (list and codes provided in eTable 1) consistent with major inpatient safety frameworks, including adverse events such as infections, complications of care, and other harms attributable to hospitalization,[20] and discharge disposition (dichotomized individually as home, rehabilitation, hospice, and other locations [e.g., discharged/transferred to short-term hospital, other inpatient care, critical access hospital, long-term care/nursing home]). Because the number of skilled nursing facility discharges was small and subject to cell suppression rules in our data use agreement, skilled nursing facility discharges were included within the broader post-acute category for statistical stability and privacy protection.

Continuous outcomes included hospitalization length of stay (LOS) and costs, which consisted of total costs, total index hospitalization costs, 30-day post-discharge costs, and inpatient readmission costs. For HaH admissions, length of stay reflects the duration of care delivered within the HaH model as captured in claims; the study period

© 2026 Vakkalanka JP et al. *JAMA Network Open*.

predates widespread use of span codes that precisely differentiate HaH days within an acute episode. Total costs were defined as all costs associated with the index hospitalization from admission date into either the HaH model or a traditional inpatient admission, and up to 30 days post-discharge based on Medicare-reimbursed payments. Cost measures reflected total allowed amounts rather than DRG base payments alone, including auxiliary billed services and cost-sharing captured in claims, and therefore could vary despite Medicare's DRG-based payment parity. In addition to the total payment from Medicare, we also included beneficiary payments (coinsurance/ deductibles) and primary payer payments (if not Medicare) for inpatient stays, observation stays, ED visits, clinic visits, ambulance services (emergency transportation to EDs, interhospital transfers, and other acute ambulance costs), residential treatment (e.g., acute rehabilitation, long-term acute care, nursing home stays), home health, durable medical equipment, and hospice care. Therefore, cost estimates in this study represent total healthcare spending observed in claims rather than Medicare program expenditures alone.

Statistical Analysis. We first descriptively assessed HaH participant hospitals included in our cohort, and categorized facilities as high versus low utilizers of HaH based on the median number of HaH admissions (median 149 admissions). This cutoff was used only for descriptive comparisons of hospital characteristics and did not represent a program maturity threshold. We tabulated utilization at the hospital-level (above and below this cut-off) by various hospital characteristics from the AHA. For dichotomous outcomes, we used conditional logistic regression to measure the association between modality of care (i.e., HaH vs traditional inpatient admissions) and each outcome within matched

© 2026 Vakkalanka JP et al. *JAMA Network Open*.

patients from the propensity score modeling and reported findings as adjusted odds ratios and 95% confidence intervals (95%CI). As our continuous outcomes (cost, LOS) were skewed, we log-transformed each outcome, used linear regression, and reported findings as the adjusted percent change (aPC) associated with HaH and 95%CIs. Due to negligible differences in standardized differences across all covariates after propensity score matching, all final adjusted models included only the modality of hospitalization (i.e., HaH vs traditional inpatient).

Sensitivity and Sub-Group Analyses. To assess baseline severity and unmeasured confounding, we developed a claims-based prognostic model for in-hospital mortality using only pre-exposure covariates and excluded modality of hospital admission (i.e., HaH vs traditional inpatient admission). We then applied this model to the matched cohorts and compared the predicted mortality risk between groups.

We tested the sensitivity of our findings within the following domains: geography (rural and urban hospitals), hospital size (200+ HaH patients and ::199 HaH patients), census region (Northeast and South), and governance (non-government and state/local hospitals). We re-estimated the propensity score within the restricted subgroup using the same covariate set applied in the main analysis. We then performed greedy matching with up to three controls per HaH admission, using a caliper of 0.1 on the propensity score and discarding observations that fell outside the caliper. Matching was implemented in SAS PROC PSMATCH using a greedy algorithm. In strata with limited common support, we allowed matching with replacement. For each subgroup, we summarized the achieved matching ratio along with the number of treated and unique control admissions in the matched analytic sample. Outcome models incorporated

© 2026 Vakkalanka JP et al. *JAMA Network Open*.

match weights where appropriate and used robust standard errors clustered on the matched set and hospital.

© 2026 Vakkalanka JP et al. *JAMA Network Open*.

**eTable 1. Hospital-related conditions and *ICD-10-CM* codes (Centers for Medicare and Medicaid/CMS)**		
**Hospital-related conditions**	**Condition number**	**ICD-10-CM Codes**
Air Embolism	02	T80.0XXA
Blood Incompatibility	03	T80 (.30XA, .310A, .311A, .319A, .39XA)
Stage III and IV Pressure Ulcers	04	L89.00-L89.32, L89.50-L89.89 (6th character = 3 or 4), L89.4, L89.9 (5th character = 3 or 4)
Falls and Trauma (includes: Fractures, dislocations, intracranial injuries, crushing injuries, burns, other injuries)	05	M99 (.10,.11,.18), S02, S12, S22, S24, S32, S42, S49, S52, S59, S62, S72, S79, S82, S89, S92
Catheter-Associated Urinary Tract Infection (UTI)	06	B37 (.41,.49), N10, N11.9, N12, N13.6, N15.1, N28 (.84,.85,.86), N30 (.00,.01), N34.0, N39.0, T83.511A, T83.518A
Vascular Catheter-Associated Infection	07	T80.21
Manifestations of Poor Glycemic Control	09	E08 (.00,.01,.10,.11), E09 (.00,.01,.10,.11), E10 (.10,.11), E11 (.00,.01,.10,.11), E13 (.00,.01,.10,.11), E15
Deep Vein Thrombosis (DVT)/Pulmonary Embolism (PE) Following Certain Orthopedic Procedures	10	I26 (.02,.09,.92,.93,.94,.99 / DVT), I82 (.401-.459, .491-.499, .4Y1-4Y9, .4Z1-.4Z9 / PE), following total knee or hip replacement: OSR9, OSRA, OSRB, OSRC, OSRD, OSRE, OSRR, OSRS, OSRT, OSRU, OSRV, OSRW, OSU9, OSUA0BZ, OSUB0BZ, OSUE0BZ, OSUR0BZ, OSUS0BZ
Iatrogenic Pneumothorax with Venous Catheterization	14	J95.811 (Iatrogenic pneumothorax), following venous catheterization: 02H (633Z, K33Z, S33Z, S43Z, T33Z, T43Z, V33Z, V43Z), 05H (033Z, 043Z, 133Z, 143Z, 333Z, 343Z, 433Z, 443Z, 533Z, 543Z, 633Z, 643Z, M33Z, N33Z, P33Z, Q33Z), 0JH63XZ

© 2026 Vakkalanka JP et al. *JAMA Network Open*.

eTable 2. Subgroup Analysis: Main Analysis, Rural Hospitals, and Urban Hospitals			
	**Main Analysis**	**Rural Hospitals**	**Urban Hospitals**
**Number of hospitals (n)**	68	6	62
**HaH (n)**	4,174	270	3,858
**Traditional Inpatient (n)**	11,697	673	10,772
**Primary Outcomes (Dichotomous)**	**aOR (95% CI)**	**aOR (95% CI)**	**aOR (95% CI)**
Mortality during hospitalization	0.09 (0.06, 0.16)	0.10 (0.01, 0.75)	0.10 (0.06, 0.17)
30-day readmission	1.07 (0.96, 1.20)	1.56 (1.02, 2.38)	1.01 (0.90, 1.13)
ED visits post-30-day discharge	0.86 (0.76, 0.97)	0.96 (0.59, 1.57)	0.87 (0.76, 0.99)
**Secondary Outcomes (Dichotomous)**	**aOR (95% CI)**	**aOR (95% CI)**	**aOR (95% CI)**
ICU escalations	0.39 (0.33, 0.48)	0.49 (0.18, 1.33)	0.39 (0.32, 0.48)
Hospital-associated complications	0.59 (0.48, 0.73)	0.76 (0.32, 1.79)	0.56 (0.45, 0.69)
Disposition			
Home	8.02 (6.93, 9.29)	10.55 (5.29, 21.04)	7.88 (6.77, 9.16)
Rehabilitation	0.09 (0.07, 0.11)	0.02 (0.002, 0.12)	0.09 (0.07, 0.12)
Hospice	0.34 (0.25, 0.45)	0.27 (0.06, 1.14)	0.33 (0.24, 0.44)
Other locations	0.53 (0.41, 0.68)	0.91 (0.35, 2.34)	0.51 (0.39, 0.67)
**Secondary Outcomes (Continuous)**	**aPC (95% CI)**	**aPC (95% CI)**	**aPC (95% CI)**
Hospital length of stay	1.23 (1.21, 1.26)	1.28 (1.18, 1.38)	1.23 (1.21, 1.26)
Costs			
Total healthcare costs	0.96 (0.94, 0.98)	0.98 (0.91, 1.06)	0.96 (0.94, 0.98)
Index hospitalization costs	1.10 (1.08, 1.12)	1.09 (1.01, 1.18)	1.10 (1.08, 1.12)
Post-discharge costs	0.65 (0.62, 0.68)	0.66 (0.55, 0.80)	0.66 (0.62, 0.69)
Inpatient readmission costs	0.97 (0.90, 1.04)	0.92 (0.73, 1.16)	0.97 (0.90, 1.05)

Reference = Traditional inpatient admission

ICU = intensive care unit; aOR = Adjusted odds ratio; aPC = Adjusted percent change; HaH = Hospital at home

© 2026 Vakkalanka JP et al. *JAMA Network Open*.

eTable 3. Subgroup Analysis: Main Analysis, Hospitals with ≥200 Beds, and Hospitals with ≤199 Beds			
	**Main Analysis**	**Hospitals with ≥200 Beds**	**Hospitals with ≤199 Beds**
**Number of hospitals (n)**	68	51	17
**HaH (n)**	4,174	3,510	591
**Traditional Inpatient (n)**	11,697	9,762	1,531
**Primary Outcomes (Dichotomous)**	**aOR (95% CI)**	**aOR (95% CI)**	**aOR (95% CI)**
Mortality during hospitalization	0.09 (0.06, 0.16)	0.10 (0.06, 0.16)	***
30-day readmission	1.07 (0.96, 1.20)	1.08 (0.95, 1.22)	1.36 (0.99, 1.87)
ED visits post-30-day discharge	0.86 (0.76, 0.97)	0.87 (0.76, 1.00)	1.16 (0.84, 1.59)
**Secondary Outcomes (Dichotomous)**	**aOR (95% CI)**	**aOR (95% CI)**	**aOR (95% CI)**
ICU escalations	0.39 (0.33, 0.48)	0.40 (0.33, 0.49)	0.28 (0.15, 0.50)
Hospital-associated complications	0.59 (0.48, 0.73)	0.54 (0.43, 0.67)	0.65 (0.35, 1.22)
Disposition			
Home	8.02 (6.93, 9.29)	7.36 (6.32, 8.57)	17.79 (9.92, 31.90)
Rehabilitation	0.09 (0.07, 0.11)	0.09 (0.07, 0.12)	0.03 (0.01, 0.09)
Hospice	0.34 (0.25, 0.45)	0.37 (0.27, 0.50)	0.25 (0.10, 0.64)
Other locations	0.53 (0.41, 0.68)	0.61 (0.47, 0.80)	0.26 (0.10, 0.66)
**Secondary Outcomes (Continuous)**	**aPC (95% CI)**	**aPC (95% CI)**	**aPC (95% CI)**
Hospital length of stay	1.23 (1.21, 1.26)	1.24 (1.22, 1.27)	1.19 (1.13, 1.26)
Costs			
Total healthcare costs	0.96 (0.94, 0.98)	0.97 (0.95, 0.99)	0.94 (0.90, 0.99)
Index hospitalization costs	1.10 (1.08, 1.12)	1.11 (1.09, 1.13)	1.07 (1.02, 1.12)
Post-discharge costs	0.65 (0.62, 0.68)	0.66 (0.63, 0.69)	0.64 (0.56, 0.72)
Inpatient readmission costs	0.97 (0.90, 1.04)	0.97 (0.90, 1.06)	1.00 (0.83, 1.22)

Reference = Traditional inpatient admission

ICU = intensive care unit; aOR = Adjusted odds ratio; aPC = Adjusted percent change

**** Model did not converge due to small sample size*

© 2026 Vakkalanka JP et al. *JAMA Network Open*.

eTable 4. Subgroup Analysis: Main Analysis and 2022 Admissions Only		
	**Main Analysis**	**2022 Admissions Only**
**Number of hospitals (n)**	68	68
**HaH (n)**	4,174	2,954
**Traditional Inpatient (n)**	11,697	8,227
**Primary Outcomes (Dichotomous)**	**aOR (95% CI)**	**aOR (95% CI)**
Mortality during hospitalization	0.09 (0.06, 0.16)	0.10 (0.06, 0.17)
30-day readmission	1.07 (0.96, 1.20)	1.06 (0.93, 1.21)
ED visits post-30-day discharge	0.86 (0.76, 0.97)	0.90 (0.77, 1.04)
**Secondary Outcomes (Dichotomous)**	**aOR (95% CI)**	**aOR (95% CI)**
ICU escalations	0.39 (0.33, 0.48)	0.31 (0.25, 0.40)
Hospital-associated complications	0.59 (0.48, 0.73)	0.52 (0.40, 0.66)
Disposition		
Home	8.02 (6.93, 9.29)	8.74 (7.34, 10.41)
Rehabilitation	0.09 (0.07, 0.11)	0.09 (0.07, 0.11)
Hospice	0.34 (0.25, 0.45)	0.34 (0.24, 0.47)
Other locations	0.53 (0.41, 0.68)	0.49 (0.36, 0.67)
**Secondary Outcomes (Continuous)**	**aPC (95% CI)**	**aPC (95% CI)**
Hospital length of stay	1.23 (1.21, 1.26)	1.23 (1.20, 1.25)
Costs		
Total healthcare costs	0.96 (0.94, 0.98)	0.96 (0.94, 0.98)
Index hospitalization costs	1.10 (1.08, 1.12)	1.10 (1.08, 1.12)
Post-discharge costs	0.65 (0.62, 0.68)	0.65 (0.61, 0.68)
Inpatient readmission costs	0.97 (0.90, 1.04)	0.97 (0.90, 1.06)

Reference = Traditional inpatient admission

ICU = intensive care unit; aOR = Adjusted odds ratio; aPC = Adjusted percent change

© 2026 Vakkalanka JP et al. *JAMA Network Open*.

eTable 5. Subgroup Analysis: Main Analysis and Admissions Excluding Arrivals from Nursing Facilities		
	**Main Analysis**	**Excluding Nursing Home Arrivals**
**Number of hospitals (n)**	68	68
**HaH (n)**	4,174	4,136
**Traditional Inpatient (n)**	11,697	11,540
**Primary Outcomes (Dichotomous)**	**aOR (95% CI)**	**aOR (95% CI)**
Mortality during hospitalization	0.09 (0.06, 0.16)	0.10 (0.06, 0.17)
30-day readmission	1.07 (0.96, 1.20)	1.11 (0.99, 1.24)
ED visits post-30-day discharge	0.86 (0.76, 0.97)	0.87 (0.77, 0.98)
**Secondary Outcomes (Dichotomous)**	**aOR (95% CI)**	**aOR (95% CI)**
ICU escalations	0.39 (0.33, 0.48)	0.40 (0.33, 0.49)
Hospital-associated complications	0.59 (0.48, 0.73)	0.59 (0.47, 0.72)
Disposition		
Home	8.02 (6.93, 9.29)	7.35 (6.33, 8.54)
Rehabilitation	0.09 (0.07, 0.11)	0.09 (0.08, 0.12)
Hospice	0.34 (0.25, 0.45)	0.36 (0.27, 0.48)
Other locations	0.53 (0.41, 0.68)	0.59 (0.45, 0.76)
**Secondary Outcomes (Continuous)**	**aPC (95% CI)**	**aPC (95% CI)**
Hospital length of stay	1.23 (1.21, 1.26)	1.24 (1.21, 1.26)
Costs		
Total healthcare costs	0.96 (0.94, 0.98)	0.97 (0.96, 0.99)
Index hospitalization costs	1.10 (1.08, 1.12)	1.10 (1.08, 1.12)
Post-discharge costs	0.65 (0.62, 0.68)	0.68 (0.64, 0.71)
Inpatient readmission costs	0.97 (0.90, 1.04)	0.99 (0.93, 1.07)

Reference = Traditional inpatient admission

ICU = intensive care unit; aOR = Adjusted odds ratio; aPC = Adjusted percent change

© 2026 Vakkalanka JP et al. *JAMA Network Open*.

eTable 6. Subgroup Analysis: Main Analysis, Hospitals in US Northeast, and Hospitals in US South			
	**Main Analysis**	**Hospitals in Northeast**	**Hospitals in South**
**Number of hospitals (n)**	68	15	29
**HaH (n)**	4,174	901	2,206
**Traditional Inpatient (n)**	11,697	2,408	5,931
**Primary Outcomes (Dichotomous)**	**aOR (95% CI)**	**aOR (95% CI)**	**aOR (95% CI)**
Mortality during hospitalization	0.09 (0.06, 0.16)	0.10 (0.03, 0.31)	0.11 (0.06, 0.22)
30-day readmission	1.07 (0.96, 1.20)	1.08 (0.84, 1.39)	1.21 (1.04, 1.42)
ED visits post-30-day discharge	0.86 (0.76, 0.97)	0.75 (0.56, 1.00)	0.96 (0.81, 1.14)
**Secondary Outcomes (Dichotomous)**	**aOR (95% CI)**	**aOR (95% CI)**	**aOR (95% CI)**
ICU escalations	0.39 (0.33, 0.48)	0.35 (0.22, 0.55)	0.47 (0.37, 0.59)
Hospital-associated complications	0.59 (0.48, 0.73)	0.68 (0.44, 1.05)	0.61 (0.45, 0.81)
Disposition			
Home	8.02 (6.93, 9.29)	7.55 (5.58, 10.22)	7.00 (5.78, 8.47)
Rehabilitation	0.09 (0.07, 0.11)	0.05 (0.03, 0.09)	0.13 (0.10, 0.16)
Hospice	0.34 (0.25, 0.45)	0.81 (0.45, 1.48)	0.27 (0.18, 0.40)
Other locations	0.53 (0.41, 0.68)	0.74 (0.46, 1.21)	0.48 (0.33, 0.70)
**Secondary Outcomes (Continuous)**	**aPC (95% CI)**	**aPC (95% CI)**	**aPC (95% CI)**
Hospital length of stay	1.23 (1.21, 1.26)	1.19 (1.14, 1.25)	1.26 (1.22, 1.29)
Costs			
Total healthcare costs	0.96 (0.94, 0.98)	0.98 (0.95, 1.02)	0.97 (0.95, 1.00)
Index hospitalization costs	1.10 (1.08, 1.12)	1.17 (1.13, 1.21)	1.09 (1.06, 1.11)
Post-discharge costs	0.65 (0.62, 0.68)	0.64 (0.58, 0.70)	0.68 (0.64, 0.73)
Inpatient readmission costs	0.97 (0.90, 1.04)	1.02 (0.86, 1.21)	0.93 (0.85, 1.03)

Reference = Traditional inpatient admission

ICU = intensive care unit; aOR = Adjusted odds ratio; aPC = Adjusted percent change

Northeast States include: Connecticut, Maine, Massachusetts, New Hampshire, New Jersey, New York, Pennsylvania, Rhode Island, and Vermont

Southern States include: Alabama, Arkansas, Delaware, District of Columbia, Florida, Georgia, Kentucky, Louisiana, Maryland, Mississippi, North Carolina, Oklahoma, South Carolina, Tennessee, Texas, Virginia, and West Virginia

© 2026 Vakkalanka JP et al. *JAMA Network Open*.

eTable 7. Subgroup Analysis: Main Analysis, Non-government Hospitals, and State/Local Hospitals			
	**Main Analysis**	**Non-government Hospitals**	**State/Local Hospitals**
**Number of hospitals (n)**	68	56	12
**HaH (n)**	4,174	3,376	715
**Traditional Inpatient (n)**	11,697	9,390	1,862
**Primary Outcomes (Dichotomous)**	**aOR (95% CI)**	**aOR (95% CI)**	**aOR (95% CI)**
Mortality during hospitalization	0.09 (0.06, 0.16)	0.06 (0.03, 0.13)	0.19 (0.09, 0.41)
30-day readmission	1.07 (0.96, 1.20)	1.12 (0.99, 1.27)	1.12 (0.84, 1.49)
ED visits post-30-day discharge	0.86 (0.76, 0.97)	0.89 (0.77, 1.02)	0.97 (0.70, 1.35)
**Secondary Outcomes (Dichotomous)**	**aOR (95% CI)**	**aOR (95% CI)**	**aOR (95% CI)**
ICU escalations	0.39 (0.33, 0.48)	0.39 (0.32, 0.49)	0.50 (0.34, 0.73)
Hospital-associated complications	0.59 (0.48, 0.73)	0.59 (0.47, 0.74)	0.40 (0.21, 0.78)
Disposition			
Home	8.02 (6.93, 9.29)	8.29 (7.04, 9.77)	6.27 (4.48, 8.78)
Rehabilitation	0.09 (0.07, 0.11)	0.09 (0.07, 0.11)	0.09 (0.05, 0.16)
Hospice	0.34 (0.25, 0.45)	0.38 (0.27, 0.52)	0.30 (0.14, 0.68)
Other locations	0.53 (0.41, 0.68)	0.50 (0.37, 0.67)	1.05 (0.60, 1.86)
**Secondary Outcomes (Continuous)**	**aPC (95% CI)**	**aPC (95% CI)**	**aPC (95% CI)**
Hospital length of stay	1.23 (1.21, 1.26)	1.24 (1.21, 1.27)	1.29 (1.23, 1.36)
Costs			
Total healthcare costs	0.96 (0.94, 0.98)	0.96 (0.95, 0.98)	0.99 (0.94, 1.04)
Index hospitalization costs	1.10 (1.08, 1.12)	1.10 (1.08, 1.12)	1.13 (1.07, 1.19)
Post-discharge costs	0.65 (0.62, 0.68)	0.67 (0.64, 0.71)	0.54 (0.48, 0.61)
Inpatient readmission costs	0.97 (0.90, 1.04)	0.98 (0.91, 1.06)	0.84 (0.72, 0.99)

Reference = Traditional inpatient admission

ICU = intensive care unit; aOR = Adjusted odds ratio; aPC = Adjusted percent change

© 2026 Vakkalanka JP et al. *JAMA Network Open*.

eFigure 1. Hospital at Home Admissions by Facility in the US (2021-2022)*

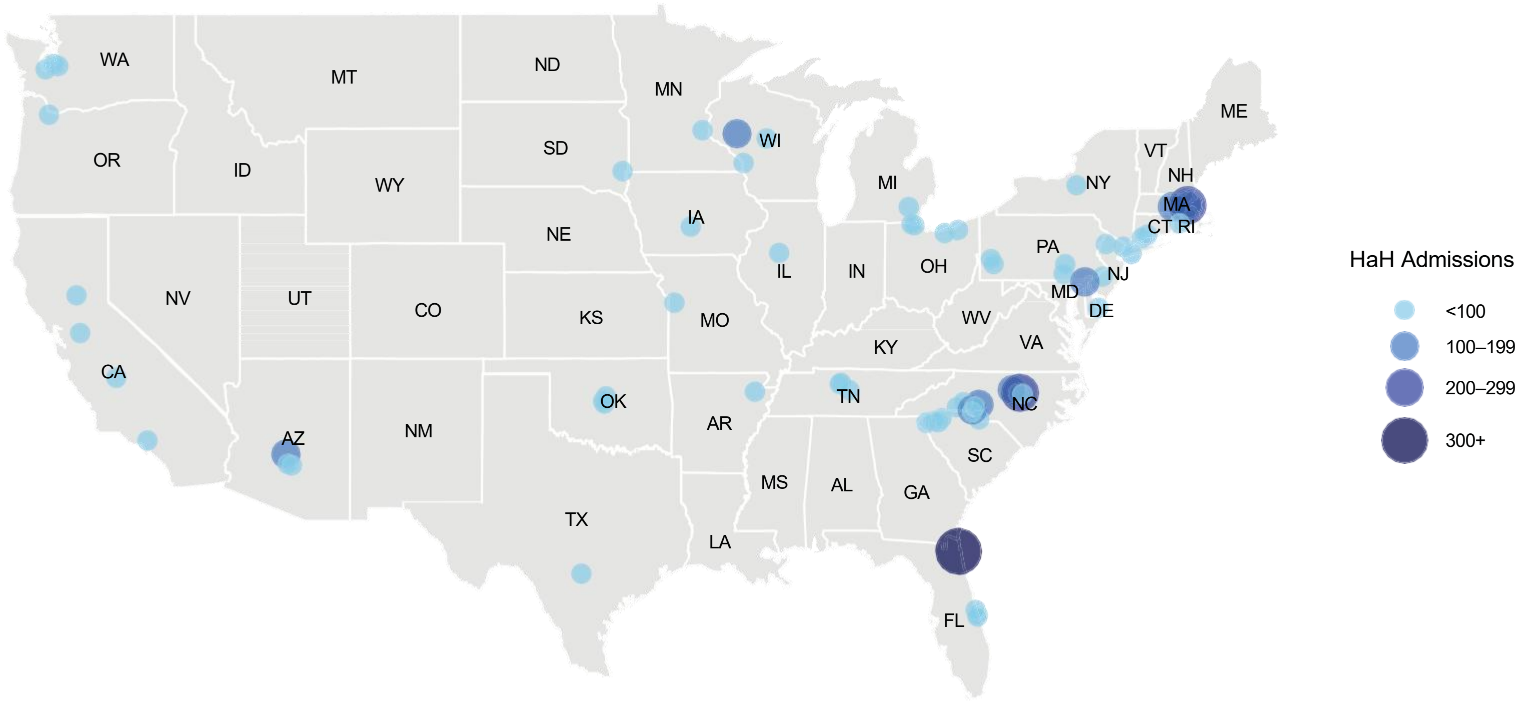

* Among 68 hospitals in our sample with at least 12 HaH admissions

© 2026 Vakkalanka JP et al. *JAMA Network Open*.

eFigure 2. Flowchart of Sample Selection and Final Analytical Cohort (Medicare, 2021-2022) Before Propensity Score Matching

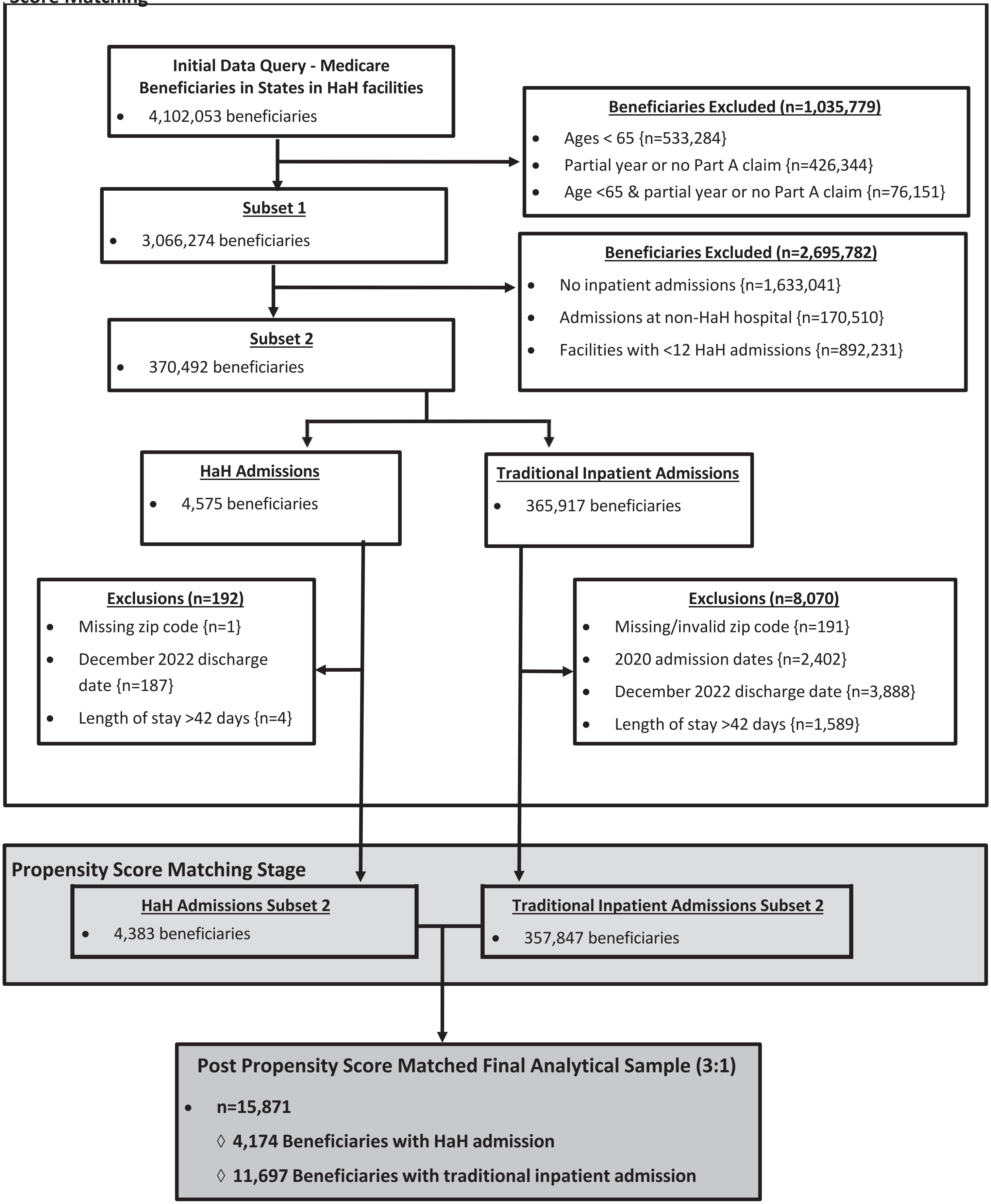

© 2026 Vakkalanka JP et al. *JAMA Network Open*.
