# Supplementary material for: Outcomes Associated With Hospital at Home vs Traditional Inpatient Stay
Source: JAMA Netw Open. 2026 May 5;9(5):e2610810. doi: 10.1001/jamanetworkopen.2026.10810 (PMC13147191; doi:10.1001/jamanetworkopen.2026.10810)
Supplement: Supplement 2. — Data Sharing Statement [file jamanetwopen-e2610810-s002.pdf]

# Data Sharing Statement

Vakkalanka. Outcomes Associated With Hospital at Home vs Traditional Inpatient Stay. *JAMA Netw Open*. Published May 05, 2026. doi:10.1001/jamanetworkopen.2026.10810

**Data**
**Data available:** No

**Additional Information**
**Explanation for why data not available:** Individual data will not be shared.
